# Electrochemical Deposition of Polypyrrole in the Presence of Silanes as Adhesion Promoters

**DOI:** 10.3390/polym15102354

**Published:** 2023-05-18

**Authors:** Andres Castro-Beltran, Clemente G. Alvarado-Beltran, Jesus F. Lara-Sanchez, Wencel de la Cruz, Felipe F. Castillon-Barraza, Rodolfo Cruz-Silva

**Affiliations:** 1 Facultad de Ingeniaria, Campus Mochis, Universidad Autónoma de Sinaloa, Fuente de Poseidón y Prol. Ángel Flores S/N, Los Mochis 81223, SIN, Mexico; andres.castro@uas.edu.mx (A.C.-B.);; 2 Departamento de Procesos de Transformación de Plásticos, Centro de Investigación en Química Aplicada (CIQA), Enrique Reyna H. 140, San José de los Cerritos, Saltillo 25294, COAH, Mexico; 3 Centro de Nanociencias y Nanotecnología de la Universidad Autónoma de México, Km 107 Carretera Tijuana-Ensenada Apdo Postal 14, Ensenada 22800, BC, Mexicofcastillon@ens.cnyn.unam.mx (F.F.C.-B.)

**Keywords:** conducting polymers, adhesion, alkoxysilanes, electrodeposition, polypyrrole

## Abstract

Polypyrrole adhesion to indium–tin oxide electrodes was improved by adding pre-hydrolyzed alkoxysilanes to the electrodeposition media. The pyrrole oxidation and film growth rates were studied by potentiostatic polymerization in acidic media. The morphology and thickness of the films were studied by contact profilometry and surface-scanning electron microscopy. The bulk and surface semiquantitative chemical composition was studied by Fourier-transform infrared spectroscopy and X-ray photoelectron spectroscopy. Finally, the adhesion was studied by scotch-tape adhesion test, where both alkoxysilanes showed a significant improvement in adhesion. We proposed a hypothesis for the improvement in adhesion, that involves the formation of siloxane material as well as in situ surface modification of the transparent metal oxide electrode.

## 1. Introduction

The field of conducting organic polymers remains promising for research, primarily due to their intriguing electrical and optical properties and high potential in a broad type of applications. Polypyrrole (PPy) is one of the most promising intrinsic conductive polymers due to its high electrical conductivity, good mechanical properties, and high chemical stability [1,2]. These characteristics make this conducting polymer useful for the fabrication of anticorrosion coatings [3,4], organic electronic devices [5,6], and electrocatalysts [7,8]. The synthesis of this polymer is carried out usually through oxidative polymerization of pyrrole. The oxidation can be carried out by many different routes, such as electrochemical [9], chemical [10], or biocatalytic methods [11]. The electrochemical polymerization was the first method developed to synthesize this polymer and remains as one of the most useful due to its simplicity. However, the poor adhesion of PPy to the substrate or electrode is usually the main problem. Several methodologies have been studied to increase electrode–PPy adhesion, such as electrode functionalization [12,13,14], optimization of the polymerization parameters [9,15], and adhesion promoters [16,17,18]. Alkoxysilanes are a group of compounds that have been used for a long time as adhesion promoters between inorganic surfaces and polymeric matrixes [19]. The use of alkoxysilanes to increase the adhesion of a conducting polymer film to the surface of an electrode has been scarcely reported. We can trace this technique to the pioneering work of Simon et al. [12], who used an oxidizable N-((3-trimethoxysilyl)propyl)pyrrole monolayer on silicon substrate simultaneously as adhesion promoter and initiator for the growth of PPy films. Y.S. Qiao et al. [20] used a silane to modify the surface of silicon, glass, and alumina substrates to increase the adhesion to in situ chemically polymerized PPy coatings. Other alkoxysilanes that have been used successfully as adhesion promoters are 3-[2-(2-aminoethylamino)ethylamino]propyl-trimethoxysilane [17,21], polydimethylsiloxane [22], and 3-aminopropyltrimethoxysilane [23]. Typically, these silanes are used to modify the surface of the substrates and the process is followed by PPy film deposition. Recently, C.M. Sougueh et al. [13] reported the use of the oxidizable N-[3-(trimethoxysilyl)propyl]pyrrole for modifying the surface of fluorine-doped tin oxide (FTO) substrates followed by electrodeposition of PPy films. These adhesives work following the classic adhesion mechanism, where the adhesive molecule consists of two moieties, one with affinity for the surface (silane, phosphonic, carboxyl, or another polar group) and another moiety that can interact strongly with PPy. In some cases, this moiety is an oxidizable group that can promote the chemical or electrochemical grafting of the adhesive to the PPy [12]. However, usually some adhesive molecules have complex architectures, making their synthesis complicated, or the PPy deposition process consists of several steps, involving the substrate modification followed by the PPy deposition. In this work, we explored a simplified method, using relatively simple silanes, such as methyltrimethoxysilane (MTMS) and N-[3-(trimethoxysilyl)propyl]aniline (TMSPA) for a two-step deposition process. We chose these silanes to assess whether oxidizable adhesives are necessary to improve the adhesion between the substrate, in this case, a fluorinated indium–tin oxide electrode, and the electrodeposited PPy. Indeed, MTMS is in principle only a surface modifier, while the TMSPA has been used before as an electrochemically active adhesive [24]. Furthermore, we studied the effects of these adhesion promoters in parameters, such as film growth, electrochemical oxidation of the monomer, and morphology.

## 2. Materials and Methods

### 2.1. Chemicals

Polypyrrole 98% reagent grade, indium–tin–oxide (ITO) coated on a polyethylenterephtalate (PET) sheet (35/sq surface resistance), N-[3-(trimethoxysilyl)propyl] aniline (TMSPA), and methyltrimethoxysilane (MTMS) were purchased from Sigma-Aldrich, Toluca, Mexico. Ethanol and hydrochloric acid were purchased from FAGALAB, Sinaloa, Mexico, and were of analytical grade and used without purification.

### 2.2. Preparation of the Monomer/Alkoxysilanes Solution for Electropolymerization

The solution was prepared from each one of the alkoxysilanes (MTMS y TMSPA), deionized water and ethanol, using a 4:2:1 *v/v* ratio, followed by 1 h of stirring to promote the alkoxysilanes hydrolysis. Simultaneously, an acidic monomer stock solution containing pyrrole (0.1 N) and hydrochloric acid (0.1 N) was prepared. To prepare the PPy–TMSPA deposition solution, 1 mL of the hydrolyzed TMSPA solution was added to 50 mL of the monomer stock solution. Similarly, the PPy–MTMS deposition solution was prepared by mixing 3 mL of hydrolyzed MTMS solution with 50 mL of stock monomer solution. These solutions were stirred further for 24 h before electropolymerization to ensure homogenization. For comparison purposes, a control PPy sample was prepared using only the stock monomer solution.

### 2.3. Electrodeposition of PPy Samples

Electrochemical polymerization of pyrrole samples was carried out in a typical three-electrode cell using a Gill-AC potentiostat from ACM instruments, Cumbria, LA11 6HH U.K. The reference electrode was an Ag/AgCl electrode, whereas the counter electrode was a platinum wire. Indium–tin oxide (ITO) coated slides were cut to 10 × 20 mm and used as working electrodes. Before the polymerization, the protective plastic film was removed from the working electrodes, and then quickly immersed in the electropolymerization bath. The deposition was started immediately to avoid electrode dissolution in the acidic media. The PPy samples were potentiostatically electrodeposited at 1000 mV vs. Ag/AgCl reference potential for 60 s.

### 2.4. Characterization

FTIR spectra were acquired in Nicolet 6700 equipment (Thermofisher, Waltham, MA, USA). PPy films were peeled off from the substrate by soaking in a 0.2 N hydrochloric acid solution, and subsequently grinded with KBr into pellets. The morphology of PPy films was observed by scanning electron microscopy (SEM) using a Jeol JSM-5300 at 15 kV (Tokyo, Japan). The thickness of the films was measured with a mechanical profilometer mark Veeco model Dektak 3 (Fullerton, CA, USA) using a diamond tip with a radius of 12.5 um. The adhesion was studied by means of the adhesion test, as indicated in the ASTM Standard D3359-02. Basically, a 3 M adhesive tape was attached to the film, and then manually detached. Thereafter, the amount of polymer peeled-off from the electrodes is considered to be proportional to the polymer-electrode adhesion. X-ray photoelectron spectroscopy (XPS) analysis was carried out using the Al Kα line in a PHI-255GR XPS equipment (Kanagawa, Japan). The XPS analysis chamber was operated at 10^−9^ Torr. The surface chemical composition (atomic %) of the samples was determined from their spectra, considering the intensities of the major peaks of each element present and their standard sensitivity factors. The molecules were optimized at density functional theory [25] and the B3LYP hybrid functional level with basis sets 6-31G*. Solvent effect was considered using the COSMO conductor-like screening model [26]. Calculations were carried out using NWChem 7.0 software [27], and the resulting wavefunction files were analyzed to obtain the electrostatic potential maps with Multiwfn 3.7 [28]. All the results were plotted with VMD 1.93 [29].

## 3. Results

### 3.1. Electrodeposition of PPy/Alkoxysilanes

**Figure 1:**
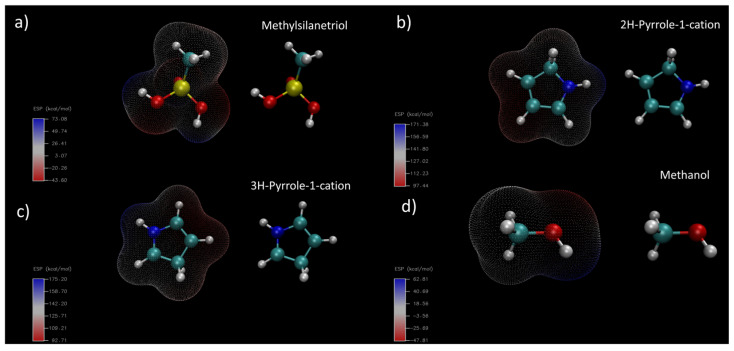
Electrostatic potential maps for the main reagents in aqueous media during PPy electrodeposition. (**a**) Methylsilanetriol, (**b**) 2H-pyrrole cation, (**c**) 3H-pyrrole cation, and (**d**) methanol.

Silanes have been used extensively as adhesion promoters in polymer science. In acidic media, methoxysilanes undergo hydrolyisis releasing methanol and resulting in silanetriol derivatives, which can attach strongly to inorganic molecules bearing hydroxyl groups on the surface, such as silica or metal oxides [30]. Before the electrodeposition of pyrrole, we hydrolyzed the silanes in order to increase their adhesion potential. Figure 1 shows the electrostatic potential surface maps of the molecules involved in the polymerization of pyrrole in acidic media in the presence of TMS. Under highly acidic condition, pyrrole molecules convert by protonation into the well-known 2H- and 3H- pyrrole cations, shown in Figure 1b,c, respectively. These molecules have a net positive +1 charge, and consequently their surfaces are mainly positive, ranging from 92 to 175 kcal/mol. These cations coexist in equilibrium with the highly negative chloride anions. On the other hand, TMS hydrolyzes resulting in methyl silanetriol (Figure 1a) and methanol (Figure 1d), which have no charge, and thus show areas with both positive and negative charges, ranging from ca. −45 kcal/mol to 62–73 kcal/mol. The surface of the molecule near the silanol group in the methyl silanetriol molecule bears a negative charge, and thus its electrostatic attraction is possible with the highly positively charged pyrrole cations, resulting in a dynamic complex in solution. Similar results are expected for the TMSPA silane molecule, and the previous results show that this molecule is able to surface modify ITO electrodes [24].

**Figure 2:**
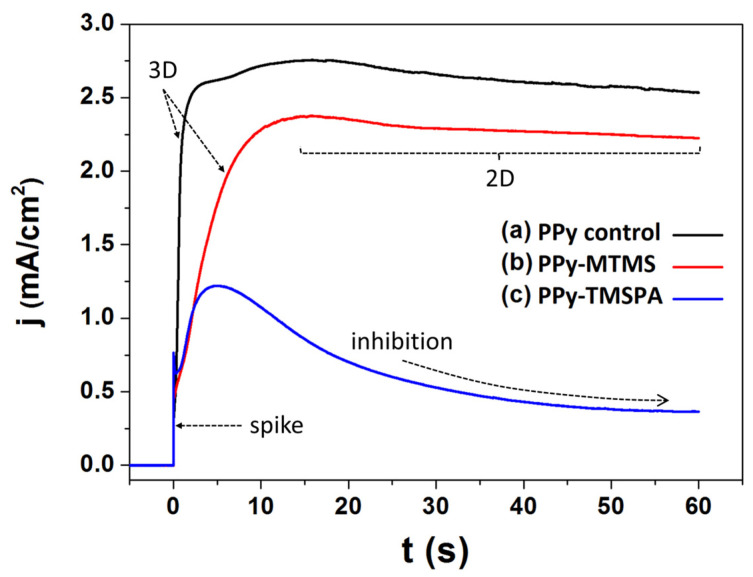
Chronoamperometry curves for the potentiostatic electropolymerization pyrrole on ITO substrates, (a) pyrrole control reaction (PPy), (b) pyrrole in the presence of methyltrimethoxysilane (PPy-MTMS), and (c) pyrrole in the presence of N-[3-(trimethoxysilyl)propyl] aniline (PPy-TMSPA).

In this work, we chose ITO electrodes for polypyrrole deposition. ITO is a transparent conductive oxide material widely used in optoelectronic devices. It dissolves slowly in acidic media, and its surface forms numerous hydroxyl groups in aqueous media, which can interact with the silanol groups of the hydrolyzed silanes. Figure 2 shows the chronoamperometric curves of polypyrrole electrodeposition in the presence of different alkoxysilanes over ITO-coated PET film electrodes. Before the deposition, there is a current spike due to the formation of the double layer. Thereafter, the PPy electrodeposition can be divided mainly in two stages. The first stage occurs during the first seconds, showing a current density growth. This indicates the oxidation of the pyrrole monomer at the electrode interface with a 3D growth, characteristic of film nucleation [24]. The nucleation refers to the initial formation of small polymer clusters or nuclei on the surface of the substrate, due to polypyrrole insolubility in aqueous media. The exact mechanism is still not well-known but it involves adsorption of the monomer on the substrate, followed by its oxidation and polymerization that leads to the formation of small clusters on the surface. During the second stage, the current usually stabilizes, indicating a 2D growth of the film [31]. Understanding the mechanisms of film 3D and 2D growth is important for optimizing the electrodeposition process and controlling the properties of the deposited film. The sample synthesized in the presence of MTMS shows a slight decrease in current density in the deposit, most likely since the alkoxysilanes partly inhibit the oxidation of the monomer, and consequently hinder the film growth. Surprisingly, the sample synthesized in the presence of TMSPA, an electrochemically oxidizable silane, shows a stronger inhibition of the oxidation, and consequently the film deposition is considerably lower.

According to Faraday’s law, the charge density is proportional to the amount of oxidized monomer, and thus the potentiostatic study (see Figure 2) shows that both alkoxysilanes act as inhibitors during pyrrole oxidative electropolymerization. This effect is evident in the reduction in charge density (Table 1). This is not surprising since silanes have been long used as anticorrosion additives to protect from oxidation [32]. The highest efficiency as oxidizer inhibitor from the TMSPA is also expected, since this in particular, has been shown to have very strong anticorrosive properties [33]. Another reason is that hydrolyzed alkoxysilanes are very well-known surface modifiers of metal oxides, and thus it is highly likely that they adsorb on the surface of the electrode, obstructing the charge transfer from the solution to the electrode.

### 3.2. Film Morphology

**Figure 3:**
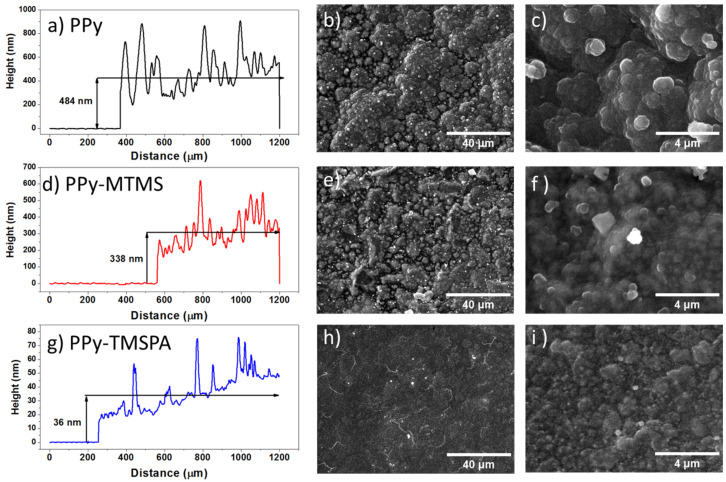
Morphology of the samples analyzed by profilometry and scanning electrical microscopy: (**a**) Height profile of PPy film, (**b**) SEM image of the PPy film surface, (**c**) high magnification image of PPy film surface, (**d**) height profile of PPy–MTMS film, (**e**) SEM image of the PPy–MTMS film surface, (**f**) high magnification image of PPy–MTMS film surface, (**g**) height profile of PPy–PTMSA film, (**h**) SEM image of the PPy–PTMSA film surface, (**i**) high magnification image of PPy–PTMSA film surface.

Topographical profiles were obtained for all samples by profilometry to measure the samples thickness, as shown in Figure 3. The average thickness of the samples is in good agreement with the charge density and the amount of polymer deposited, as measured by the thickness of the film. This suggests that the alkoxysilanes interference occurs during the oxidation stage of pyrrole and not during the growth of the film by polymerization on the substrate. The absence of polymer colloids in the electrolyte also indicates that most of the polymer resulting from the oxidation of the monomer attaches to the electrode. Therefore, the PPy sample has a greater thickness compared with the PPy–MTMS sample, whereas the thickness of PPy–TMSPA sample was smaller than those of the previous two samples (see Table 1).

**Table 1:** Charge density and thickness of the samples prepared.

Sample Label	Alkoxysilanes	Charge Density (mC/cm^2^)	Thickness (nm)
PPy	None	397	484
PPy–MTMS	MTMS	213	338
PPy–TMSPA	TMSPA	69	36

The effect of the alkoxysilanes on the morphology of the PPy film was studied using scanning electron microscopy. Figure 3 shows the low and high magnification images of the PPy, PPy–MTMS, and PPy–TMSPA. The PPy film sample (Figure 3b,c) shows a surface morphology with high roughness, and globular aggregates in the surface which are characteristic of electrodeposited PPy [9]. Adding silanes to the reaction media produces film samples with smoother surfaces. For example, when MTMS was added, the resulting PPy–MTMS sample (Figure 3e,f) still shows an irregular surface. However, it is smoother than the previous PPy sample, whereas the PPy–TMSPA film sample displays an almost flat morphology at low magnification (Figure 3h), but at higher magnification (Figure 3i), it can be observed that the morphology is still rough and reveals the growth of small globular features. Both SEM and profilometry show that PPy electropolymerization is hindered in the presence of these alkoxysilanes due to reduced oxidation of the monomer, resulting in less polymer deposition compared to the control sample (PPy). We observed that, in all cases, a lower deposition rate led to a smoother surface, which suggests more ordered film growth due to slower polymerization [15]. However, not all morphological changes can be ascribed solely to the slower polymerization rate, as the formation of the polymer film is a complex process that depends not only on the monomer oxidation rate, but also on many factors, such as composition of the electrolyte, chemical reactivity of the species, nucleation of the polymer on the electrode, and other factors, such as density, viscosity, and pH of the reaction media.

### 3.3. Spectroscopic Characterization

**Figure 4:**
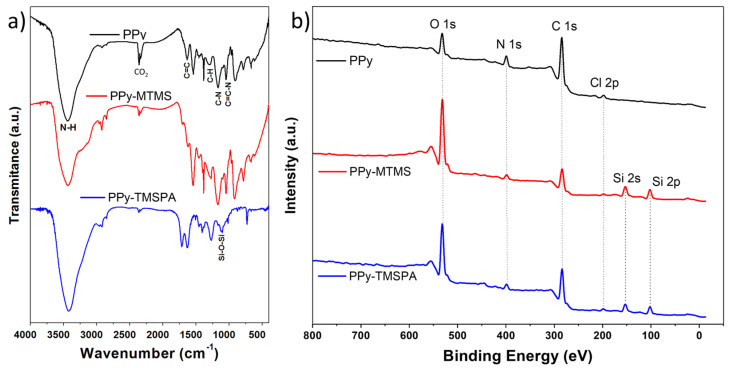
(**a**) Fourier-transform infrared spectroscopy study and (**b**) X-ray spectroscopy of polypyrrole films. From top to bottom, PPy sample, PPy–MTMS sample, and PPy–TMSPA sample.

We studied the PPy films by FTIR and XPS spectroscopic techniques in order to detect and quantify the presence of the alkoxysilanes. Figure 4a shows the FTIR spectra of pure PPy films and the PPy films prepared in the presence of MTMS and TMSPA. The FTIR spectra of the films show a prominent peak at 3440 cm^−1^ due to the stretching vibration of the N–H bond in polymer backbone [34]. The PPy sample shows the typical peaks of pyrrole, such as the 1635 cm^−1^, 1541 cm^−1^, and 1460 cm^−1^ peaks attributed to the stretching vibration of the C=C, C–C, and C–N bonds of the fundamental vibrations in the pyrrole ring, respectively. The peaks at 1288 cm^−1^ and 1045 cm^−1^ are assigned to a combination C–H in-plane ring bending and the deformation of the five-membered ring that contains the C=C–N deformation [35,36]. Moreover, the peak at 1168 cm^−1^ is related to C–N stretching wagging vibrations, the peak at 1381 cm^−1^ is associated with C–N stretching vibration [37], and the peak at 912 cm^−1^ is assigned to C=C in-plane bending vibrations of the pyrrole ring [38].

The PPy spectrum peaks correspond well to the previously reported PPy films prepared by electrodeposition [39]. The presence of alkoxysilanes can be confirmed by additional absorption bands in the samples prepared in the presence of these adhesion promoters. For example, the FTIR spectrum of PPy–MTMS shows the methyl peaks at 2850–2920 cm^−1^ due to –CH2 symmetric and asymmetric stretching, as well as increased adsorption in the Si–O stretching region (1110 cm^−1^) that overlaps with the previously discussed PPy peaks. [40]. Similarly, the FTIR spectrum of PPy–PTMSA shows two peaks at 1620 and 1710 cm^−1^ assigned to the N–H bond of the aromatic ring of the alkoxysilane. In the low frequency region, the peak at 730 cm^−1^ assigned to N–H wagging is also characteristic of the TMSPA [33]. Figure 4b shows the wide scan XPS spectra of PPy, PPy–MTMS, and PPy–TMSPA that confirm the presence of alkoxysilanes for the samples prepared in the presence of these compounds. XPS survey spectra of all samples showed the presence of main peaks of nitrogen (N 1s) and carbon (C 1s) which are characteristic of PPy, the main peak of oxygen (O 1s) which is due in part to O_2_ molecules adsorbed in the surface of the sample by contact with the environment. Chlorine (Cl 2p) is attributed to the electrolyte used (HCl) which works as counterion (doping agent) of the PPy. These peaks were present in all the samples, however, the PP–MTMS and PPy–TMSPA samples displayed the peaks of silicon (Si 2p and Si 2s), suggesting the modification of the film with alkoxysilanes, in agreement with the FTIR analysis. Similar XPS results were obtained in the work carried out by Cossement et al. [31], after electropolymerization of pyrrole in the previously modified electrodes with organosilanes. Although not fully understood, the process of silane deposition during electrodeposition may entail the adsorption of silane on both the ITO electrode and the PPy clusters. Additionally, it may involve the formation of siloxane chains which become integrated into the developing PPy film. Semiquantitave surface chemical analysis was carried out by integrating the core-level spectra of the major peaks (O 1s, N 1s, C1s, and Si 2p Cl 2p), and dividing the resulting area by their corresponding sensitivity ratios [41]. The results are shown in Table 2.

As expected, the PPy atomic concentration obtained matches the stoichiometry of PPy (C_4_N). For samples PPy–MTMS y PPy–TMSPA, the change in stoichiometry compared to the PPy is attributed to the formation of new material, mainly clusters of silicon on both the surface and bulk of the PPy film since alkoxysilanes are well-known for their self-crosslinking and polymerization properties. This silicon is incorporated into the PPy as a siloxane network as suggested by the binding energy of the Si 2p peak (102.4 eV), which is characteristic of these types of materials [33]. This is supported by the simultaneous increment in the O 1s peak, and the Si/O ration that matches the SiO_2_ composition. Similar results have been observed when adding silanes to thermoplastic polymers [42] or when alkoxysilanes are hydrolyzed into highly crosslinked organosilane networks. The formation of a siloxane network might be influenced by several factors, including the type and concentration of the silane molecules, Ph, temperature and composition of the electrolyte, and the properties of the electrode surface. Controlling these factors can be important for optimizing the formation of a siloxane network and achieving the desired properties of the resulting hybrid PPy films.

### 3.4. Adhesion Test

**Figure 5:**
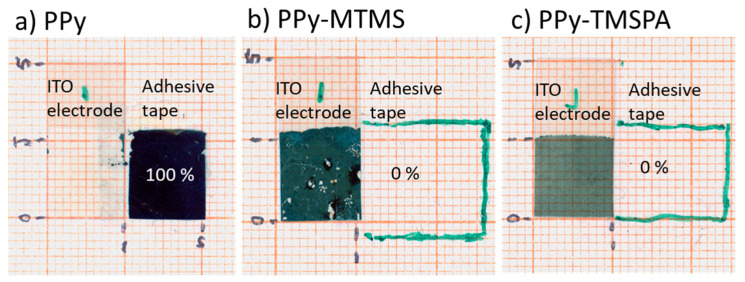
Scotch-tape adhesion test of polypyrrole films: (**a**) PPy films control sample, and (**b**) PPy–MTMS and (**c**) PPy–TMSPA films. In samples (**b**,**c**), the boundary of the transparent adhesion test was outlined with green to ease its observation.

The adhesion test is a simple but very pragmatic and reliable test to compare the relative improvement in adhesion [24]. Figure 5 shows all PPy samples (left) and the adhesive tape (right) after the adhesion test. We observed that the PPy sample was completely detached (Figure 5a), leaving a clean substrate, which indicates a very poor adhesion between the ITO substrate and PPy film, a problem common in PPy films prepared by electrodeposition on ITO [16].

**Table 2:** Element composition and silicon–oxygen ratios of PPy, PPy–MTMS, and PPy–TMSPA obtained by the XPS.

Sample	Element Composition (%)					Ratio Si/O
	O 1s	C 1s	Si 2p	N 1s	Cl 2p	
PPy	10.0	69.9	-	17.5	2.6	-
PPy–MTMS	40.6	30.7	21.1	3.9	3.7	0.52
PPy–TMSPA	25.2	50.1	13.4	8.5	2.8	0.53

For the sample PPy–MTMS, it was observed that small patches accounting for ca. 10% of the PPy film were removed from the ITO substrate (Figure 5b), which indicated a slight improvement in adhesion between the electrode and the polymer film. Furthermore, the sample PPy–TMSPA had a perfect adhesion to the sample, and the film withstood perfectly the adhesion test (Figure 5c). We believe that the significant improvement in the adhesion is due to the in situ modification of the electrode with the silane, as well as the formation of a crosslinked network of silanes that bond the PPy to the modified electrode. Our electronic calculations show that MTMS can associate with the pyrrole cations by electrostatic attraction, and thus they are highly likely adsorbed into the bulk during the pyrrole polymerization. Several studies have also shown that ITO substrates form hydroxyl groups after hydration in aqueous media, providing anchoring points to the silane adhesion promoters.

Improved adhesion of the PPy films can have a positive impact on their application lifespan. These films are less likely to delaminate or peel off over time, which can lead to premature failure of devices, such as electrochromic films or optical sensors. In addition, PPy coatings with good adhesion can provide better protection against corrosion, abrasion, and other types of wear and tear. When the PPy is strongly adhered to the substrate, it forms a barrier that can prevent water, chemicals, and other corrosive agents from reaching the underlying material [3]. This can help in prolonging the lifespan of the substrate and reducing the need for costly repairs or replacements. The improved adhesion can also enhance the morphological features of the PPy coating. In this case, it is possible that due to the good adhesion, the PPy films resulted in more homogeneous nucleation that resulted in smoother, more uniform films compared to the PPy control. This can be particularly important to reduce fouling in anticorrosive coatings, one of the targeted applications of these films.

## 4. Conclusions

The adhesion of PPy films to ITO electrodes was significantly improved using a relatively simple method, which consists of adding pre-hydrolyzed metyltrimethoxysilane and N-[3-(trimethoxysilyl)propyl]aniline to the electropolymerization media. Indeed, there was a reduction in the polymer growth rate, but on the other hand, the films were smoother. This methodology is simple, it does not require previous electrode modification steps, and can possibly be used with other substrates, such as metallic surfaces, due to the great adhesion spectrum of alkoxysilane adhesion promoters. Further work is needed to understand the effects of these adhesion promoters on the electrochemical and optical properties of the resulting polypyrrole films.

## Data Availability

Original data from this work are available under request.
